# Design and optimization of ganciclovir solid dispersion for improving its bioavailability

**DOI:** 10.1080/10717544.2022.2083723

**Published:** 2022-06-08

**Authors:** Dalia A. Gaber, Manar A. Alnwiser, Nadia L. Alotaibi, Rawan A. Almutairi, Sumaih S. Alsaeed, Siham A. Abdoun, Amal M. Alsubaiyel

**Affiliations:** a Department of Pharmaceutics, College of Pharmacy, AL-Qassim University, Qassim, KSA; b Department of Quality Control & Quality Assurance, Holding Company for Biological Products and Vaccines, Cairo, Egypt; c College of Pharmacy, AL-Qassim University, Qassim, KSA

**Keywords:** Gancyclovir, cyclodextrin, shellac, solid dispersion, nanoparticles, factorial design

## Abstract

Development of new approaches for oral delivery of an existing antiviral drug aimed to enhance its permeability and hence bioavailability. Ganciclovir (GC) is an antiviral drug that belongs to class III in biopharmaceutical classification. The encapsulation of poorly absorbed drugs within nanosized particles offers several characteristics to drug due to their acquired surface properties. In the following study, the solvent evaporation technique was used to incorporate GC, within elegant nanosize particles using cyclodextrin and shellac polymers for enhancing its permeability and release pattern. Formulation variables were optimized using 2^3^ full factorial design. The prepared formulations were assessed for yield, particle size, content, and micromeritics behavior. The optimized formula (F6) was identified through differential scanning calorimetry and Fourier transform infrared. *In vitro* release and stability were also assessed. Pharmacokinetic parameters of optimized nano GC solid dispersion particles (NGCSD-F6) were finally evaluated. The optimized formula (F6) showed a mean particle size of 288.5** **±** **20.7** **nm, a zeta potential of about 23.87** **±** **2.27, and drug content 95.77** **±** **2.1%. The *in vitro* drug release pattern of F6 showed an initial burst release followed by a sustained release over the next 12** **h. The optimized formula showed accepted stability upon storage at room and refrigerator temperatures for 6** **months with good flowing properties (Carr’s index** **=** **18.28** **±** **0.44). *In vivo* pharmacokinetic study in rabbits revealed 2.2 fold increases in the bioavailability of GC compared with commercial convention tablets. The study affords evidence for the success of the solid dispersion technique under specified conditions in improvement of bioavailability of GC.

## Introduction

**Figure 1.**
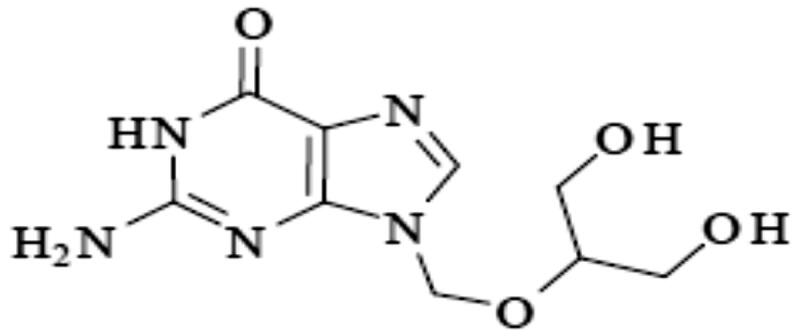
Chemical structure of ganciclovir.

Ganciclovir (GC) or 9-(1,3-dihydroxy-2-propoxym ethyl) guanine is a nucleoside analogue of guanosine, a homologue of acyclovir (Figure 1; Hong & Wang, 2018). Its log p equal −1.7, and it has a molecular weight equal to 255.2** **g/mol (Patel et al., 2016). GC is the first antiviral drug showed an accepted effectiveness in the treatment of cytomegalovirus infections in humans (Kiroula & Negi, 2016; Galar et al., 2021). It can inhibits both the herpes viruses and the transformation of normal cord blood lymphocytes by Epstein–Barr virus (Hong & Wang, 2018). The antiviral activity of GC outcomes from its transformation to the triphosphate forms which able to inhibit viral DNA polymerases by competitively, inhibiting the incorporation of deoxyguanosine triphosphate into elongated viral DNA (Märtson et al., 2021). GC has the ability to inhibit the replication of the viral DNA but does not eliminate the virus from the tissues; so long term therapy is required (Wang et al., 2018; Märtson et al., 2021). GC belongs to class III in biopharmaceutical classification system (Asasutjarit et al., 2020). The oral bioavailability of the drug is about 5% (Öztürk-Atar et al., 2019). The main reason for the low bioavailability of GC is the poor permeability through the gastrointestinal tract epithelia (Galar et al., 2021). Hence, a great interest was paid to develop a delivery system for GC which able to enhance the absorption of GC. Different delivery systems for GC have been developed, such as solid lipid NPs of GC, GC-loaded albumin NPs, and liposome-encapsulated GC (Wang et al., 2018; Öztürk-Atar et al., 2019; Asasutjarit et al., 2020). Oral drug delivery systems provide the highest patient satisfaction due to self and painless use (Yeung et al., 2020). Many researches have been recently published aimed to enhance the bioavailability and improve the sustainability of oral dosage forms of GC (Yeung et al., 2020). Many difficulties are opposite oral dosage forms of GC due to its low permeability through GIT epithelia, and hence low bioavailability (Merodio et al., 2001; Sarbajna et al., 2011).

**Figure 2.**
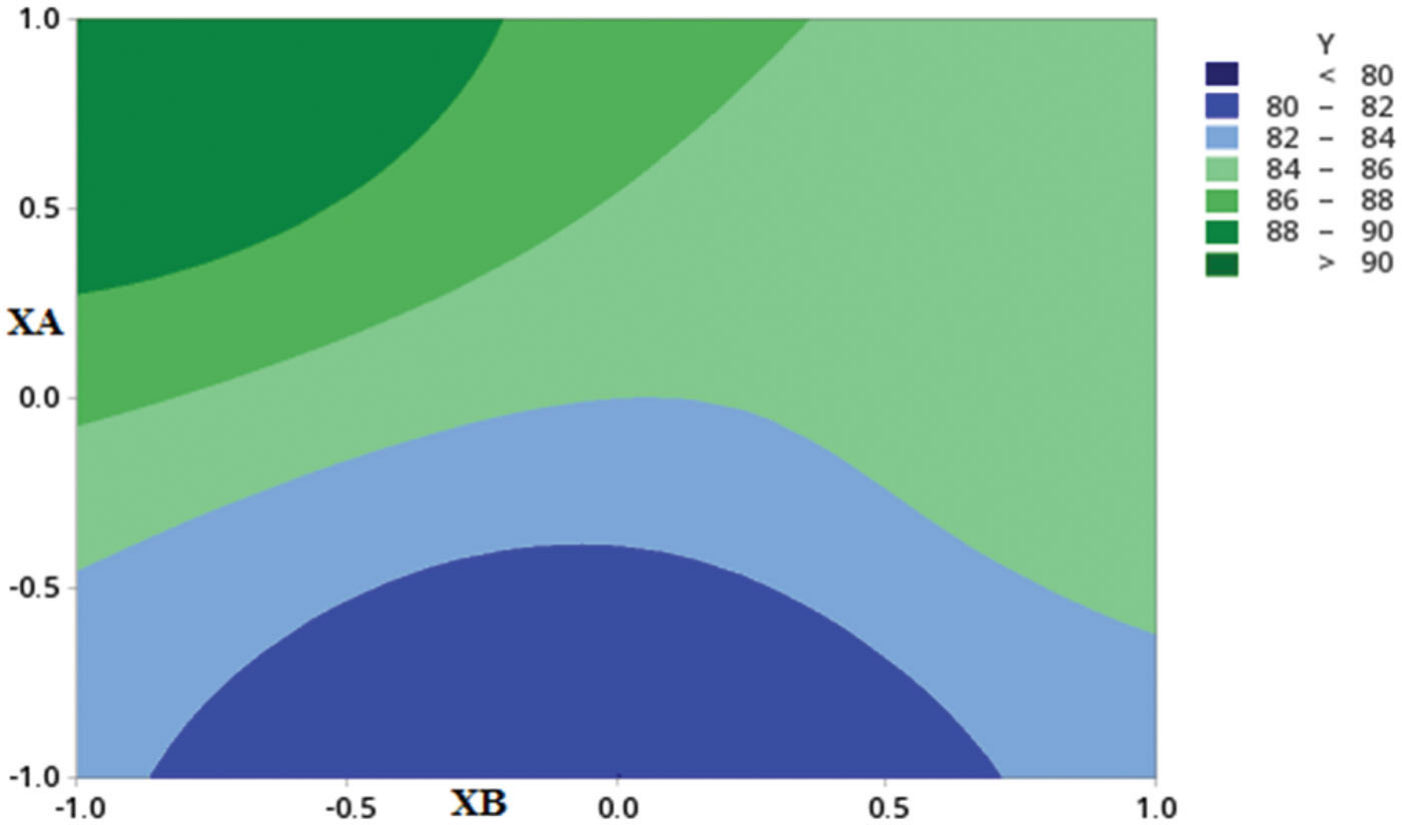
Contour plot showing the effect of CDX concentration (XA) and SHC concentration (XB)) on %DC.

The approaches done to advance the rate of absorption of such drugs are based on enhancing their passage through the intestinal wall (Sarbajna et al., 2011). Many techniques are existing to improve the permeability of low permeable drugs (Desai et al., 1996). For example, the use of prodrug form, re-crystallization of the drug, micro emulsification, electrospray, and solid dispersion method using biodegradable polymers (Gill et al., 2010; Ting et al., 2018; Sharma et al., 2019; Raza et al., 2021). Solid dispersion is a well-established technique used in enhancing the absorption of poorly permeable drugs (Raza et al., 2021). Encapsulation of drugs in nanosize solid dispersion structures demonstrates many advantages for the encapsulated drugs over free ones (Ting et al., 2018). It provides a larger contact area for drug absorption and hence better bioavailability (Ghosh et al., 2019). The properties of the solid dispersion structures can be further modified to improve the release profiles, penetration behavior, and thus achieving higher cellular uptake, lower doses, less drug resistance, less adverse reactions, and shorter treatment duration (Ghosh et al., 2019; Ricarte et al., 2019; Tran et al., 2019; Raza et al., 2021). Biodegradable polymer carriers such as shellac, hydroxyl propyl methyl cellulose, cyclodextrin, and urea, are widely used in drug encapsulation techniques (Jaglal et al., 2021). Drug encapsulation within biodegradable polymers can be advantageous to combine both the bioavailability and stability of drugs (Vidyadhara et al., 2011; Jaglal et al., 2021). Adjusting of process to formulate solid dispersion structures in a nanosize range provides larger surface area, longer shelf-life, and improved bioavailability (Tran et al., 2019). The process of solid dispersion is done by solvent evaporation, melting, and spray drying methods (Jaglal et al., 2021). Recent work has demonstrated that encapsulated particles designed based on solid dispersion technique improved the absorption of drugs through GIT (Vidyadhara et al., 2011). Bali et al encapsulated ceftriaxone using blood serum albumin for enhancing its bioavailability (Bali et al., 2018). Mehta et al. prepared solid dispersion of Glimepiride by solvent evaporation method for improving its oral absorption (Mehta et al., 2009). Shellac (SHC) is a natural animal-produced resin and wax blend which is used as a cement, a film-forming material, complexing, and solid dispersing agent (Limmatvapirat et al., 2008). Cyclodextrin (CDX) is a natural cyclic oligosaccharide that is widely used in complexing, kneading, co-precipitation, and solid dispersion process to increase the permeability of poorly permeable drugs (Abarca et al., 2016). The ongoing study aimed to encapsulate GC in the form of nanosized particles using the solid dispersion method. Two natural polymers SHC and CDX will be used at two levels. The prepared formulae were evaluated for the yield, drug content, micromeritics characters, thermal behavior, and in vitro release pattern to select the optimized formulation. The optimized formula was evaluated based on the following tests: scanning electron microscope, Fourier transform infrared (FT-IR), differential scanning calorimetry (DSC), *in vitro* release, stability, and pharmacokinetics in rabbits in comparison to commercial tablets.

## Materials and method

### Materials

Ganciclovir (GC) was obtained from Fresenius Kabi (Bad Homburg, Germany). 2- Hydroxypropyl-ß-cyclodextin (CDX) was purchased from Oxford Laboratory Chemicals (Mumbai, India), shellac (SHC) was kindly obtained from Bad Homburg, Germany. Analytical grade of disodium hydrogen phosphate, potassium phosphate monobasic, hydrochloric acid (HCL), ortho-phosphoric acid, and absolute ethanol was obtained from El-Nasr Pharmaceutical Chemical Co, (Cairo, Egypt). HPLC grade ammonium acetate and methanol were purchased from Cornell Lab (Cairo, Egypt). Acyclovir was purchased from Sigma-Aldrich (Saint-Louis, MO, United States). Hard gelatin capsules of size 3 were purchased from El-Nasr Pharmaceutical Chemical Co. (Cairo, Egypt). All other chemicals were of analytical grade and were used as received without further modifications. Double-distilled water was used for the experiments.

## Methods

### Design of experiment

The experiment was designed based on a 2^3^ factorial design to define the correlation between basic process parameters (BPs) and basic quality aspects (BQs). For enhancing the drug bioavailability; vital BQs include the highest yield, highest drug content percentage, highest entrapment efficiency, minimal size, and reasonable zeta potential were considered. The basic process parameters (BPs) were: SHC concentration (X_a_), CDX concentration (X_b_), and GC concentration (X_c_) were explored at two levels represented as 0 for the low level and 1 for the high level as shown in Table 1.

**Table 1.** Full factorial design levels (2^3^) and independent variables.

BPs	Concentration (%)	Minimum (%)	Maximum (%)	Minimum code	Maximum code
X_a_	SHC	1	2	0	1
X_b_	CDX	1	2	0	1
X_c_	GC	0.5	1	0	1

Eight formulae were prepared each with 3 runs, following the design and characterized for six aspects namely; Q1: Drug content (DC), Q2: Entrapment efficiency (% EE), Q3: Yield percentage, Q4: Particle size (PS),Q5: zeta potential (ZP), and Q6: Poly dispersity index (PDI).

The first-order regression equation was introduced as follows:

$$Y={ß}_{0}+{ß}_{1}X_{a}+{ß}_{2}X_{b}+{ß}_{3}X_{c}+{ß}_{4}X_{a}X_{b}+{ß}_{5}X_{a}X_{c}+{ß}_{6}X_{b}X_{c}+{ß}_{7}X_{a}X_{b}X_{c}$$

Where:

Y is the dependent variable (BQs).

X_a_, X_b_, X_c_ are the independent variables (CPPs).

β_0_ is the arithmetic mean of the eight runs.

β_1_, β_2_, β_3_ are the linear coefficients.

β_4_, β_5_, β_6_ are the coefficients of interaction between the two BPs.

β_7_ is the coefficient of interaction between the three BPs.

Based on the optimal level of each independent variable, the optimized formula was determined. FT-IR, DSC, flow properties, scanning electron microscope image, *in vitro* release, stability, and pharmacokinetics in rabbits were attributed to the optimized formula.

### The preparation of nanosize particles of GC using solid dispersion method

Nano solid dispersions of GC were prepared by a solvent evaporation technique (Chavan et al., 2020). The weighed amounts of GC and the carriers (SHC or CDX) were dissolved in 10** **ml of absolute ethanol in a round bottom flask. The solvent was evaporated using a Heidolph rotavap (Schwabach, Germany), rotated at 200** **rpm at room temperature under 750** **mmHg pressure until complete drying. The solvent was evaporated, leaving the GC solid dispersion (Chavan et al., 2020). The obtained sediment was re-suspended in deionized water and dried by lyophilization using Tray Lyophilizer (The VirTis Company, Inc. Gardiner, NY, United States). Lyophilized formulae were kept in a desiccator at room temperature until further studies.

## Characterization of the prepared nano ganciclovir solid dispersion (NGCSD)

### The yield percentage

The percentage of the yield for each formula of NGCSD was calculated by dividing the total weight of the dry powder of NGCSD by the total weights of starting materials (GC and the polymer) according to the following equation (Reginald-Opara et al., 2015):

$$\mathrm{Yield}\%=\frac{Wp\;}{Wt}*100$$

Where *Wp*: Weight of dry powder of NGCSD

*Wt*: Total weights of starting materials

### The percentage of GC content (DC%)

The actual content of GC was determined by dispensing five mg of dry NGCSD in 10 ml of phosphate buffer (pH 7.4) in a vortex mixer, then stirring the mixture for 2** **h. The obtained dispersion was then filtered using 0.45** **μm Millipore filter papers to remove any residues, if any; 0.2** **ml of the solution was withdrawn a diluted up to 10** **ml with phosphate buffer (pH 7.4) and measured spectrophotometrically at 254** **nm. Blank samples were taken from plain solid dispersion particles which were treated in the same manner to a cancel any interference from polymers at this wavelength (Noolkar et al., 2013).

### The percentage of entrapment efficiency (%EE)

The percentage of entrapment efficiency of GC in NGCSD was determined by suspending 5 mg of dry NGCSD in 5** **ml buffer (pH 7.4) and centrifuging the mixture for 30** **min at 3000** **rpm, the amount of un-entrapped GC in the clear supernatant after centrifugation was determined (Wallace et al., 2012). The supernatant was filtered through a Millipore filter (0.45** **μm) and the absorbance of the supernatant was measured using UV spectrophotometer at λ_max_ 254** **nm after proper dilution with phosphate buffer (pH 7.4).

The %EE was calculated as the following:

$$\%\mathrm{EE}=\frac{\text{The amount of entrapped drug}}{\text{Total amount of the drug}}\;*\;100$$

### Particle size (PZ) and polydispersity index (PDI)

PZ and PDI of the prepared NGCSD were determined using dynamic laser light scattering (Zetasizer Ver. 5.11 Malvern). One milligram from each NGCSD formulae were dispersed in a 10** **ml of deionized water for measurement (Noolkar et al., 2013).

### Zeta potential (ZP) of NGCSD formulations

Freshly prepared samples were used for ZP measurements using photon correlation spectroscopy instrument (Malvern Instruments Limited, United Kingdom) at 25** **°C (Wallace et al., 2012).

### Evaluation and characterization of the optimal formula

Based on the results of the previous tests and the factorial design test F6 was showed the optimum results and was selected for further evaluation.

### Flow properties

The flow properties of the prepared sample were assessed for ensuring effective mixing and capsule filling. The flowing characteristics including the angle of repose, bulk density, tapped density, Carr’s index, and Hausner’s ratio for the dried F6-NGCSD sample were considered (Shaikh et al., 2011).

### Angle of repose (θ) test

The frictional force between NGCSD particles was measured using angle of repose test using the funnel method, and it was calculated as following (Kamalakkannan et al., 2013):

tan ⁡θ=2HR

Where θ represents the angle of repose, *H* is the height of the powder cone in centimeters, and *R* is the diameter of the cone of powder in centimeters.

### Hausner’s ratio (HR) test

Hausner’s ratio was calculated for selected formula (NGCSD) particles by placing 5** **gm of powders in a 10** **ml dry clean cylinder. The bulk volume (*Vb*) and the tapped volume (*Vt*) were recorded and the average of three determinations was calculated and used in the following equation (Kamalakkannan et al., 2013).

$$\mathrm{HR}=\frac{Vb}{Vt}$$

### Carr’s index

Carr’s index is one of the parameters that is used to expect the flowability of the powder.

Carr’s index of the prepared GC nono-solid dispersion was calculated by the following equation (Kamalakkannan et al., 2013).

$$\text{Carr’s}\;\text{index}=\frac{\text{Tapped density}-\text{Bulk density}}{\text{Tapped density}}*100$$

### Differential scanning calorimetry (DSC)

DSC of GC, polymers (SHC, CDX), physical mixtures at a ratio of 1:1, and dried NGCSD-F6 was performed using Differential Scanning Calorimeter ((Mettler Toledo, Switzer). Five milligrams of each sample were heated from 50** **°C to 300** **°C at a heating rate of 10** **°C/min.

### Fourier transform infrared spectroscopy (FT-IR)

Defining the probable interaction between GC and polymers in F6-NGCSD was revealed. FT-IR study was conducted using FTIR Spectrophotometer study (Shimadzu 1800, Japan). Sample powder was blended with Potassium bromide powder and hydrostatic press made the pellet. The test was conducted over a scanning range of 400 to 4000** **cm^−1^.

### Scanning electron microscope (SEM)

Examination of the optimum formula (NGCSD-F6) morphology was done using SEM (Metler Toledo, Tokyo, Japan). One milliliter of the prepared NPs suspension was fifty fold diluted with deionized water and sonicated 10** **min. One drop of the diluted sample was dripped in individual stubs and coated uniformly with gold and soft imaging was performed.

### Stability study

The stability of the optimized formula under different temperature and humidity conditions was studied for six months according to ICH Q1A (R2) guidelines. Three 10** **ml amber glass bottles were filled each with 5 g of freshly prepared NGCSD particles and closed with air-tight closures; then they were stored at three different conditions; the first bottle was stored at refrigerated temperature (4** **°C** **±** **2** **°C) and humidity (40% ± 10% RH), the second bottle was kept at room temperature (25** **°C** **±** **0.5** **°C/40% ± 10% RH), the last one was placed in a desiccator with a relative humidity adjusted at 70** **±** **10% by saturated sodium chloride solution and stored in hot air oven (Metler Toledo, Tokyo, Japan) adjusted at 40** **°C** **±** **5** **°C. Samples were taken initially and after one, two, three, and six** **months and evaluated for the change in appearance, particle size, zeta potential, polydispersity index, Carr’s index, and drug content (Pathak & Pattnaik, 2018).

### *In vitro* release pattern of GC from optimized formula packed in hard gelatin capsules

Weights of optimized freeze-dried NGCSD particles equivalent to 250** **mg of GC were filled in hard gelatin capsules size 3. The amounts of GC released from the selected formula as a function of time were studied using USP type II apparatus. Capsules filled with the selected formula were added to 900** **ml of 0.1** **N HCL solution (pH 1.2) at 37** **°C** **±** **5** **°C for 2** **h then the media was shifted to pH 7.6 using phosphate buffer and samples were taken up to 24** **h. The paddle speed was adjusted at 75** **rpm. Five milliliters of samples were withdrawn at each time point, filtered through a 0.45** **µm filter and assayed for GC content using UV spectrophotometer at 254** **nm. Fresh medium was added to the dissolution medium to keep sink conditions. The test was repeated three times (*n*** **=** **3) and the average of the results was taken (Soni et al., 2017).

### Kinetic study

The release data were studied using different kinetic models which include zero-order, first-order, Higuchi diffusion, and Korsmeyer–Peppas models (Maheen et al., 2020). The highest coefficient of determination (R2) model was considered to represent the drug release mechanism.

### *In vivo* pharmacokinetic study in rabbits

Eighteen male New Zealand rabbits (≈1.5** **kg) were used in this study in agreement with the stated principles of animal care published by the European center for the validation of alternative methods (NIH publication No. 85–23, revised 1985), and with the ethical approval of the Scientific Committee of Faculty of Pharmacy, Al-Qassim University. The rabbits were equally and randomly divided into three groups. No food was served to the rabbits with free access to water 12** **h before the experiment (Maghrabia et al., 2019). Each rabbit in the first group was given one capsule of the selected formula equivalent to 250** **mg/kg of GC orally via gastric intubation. Tablets of the marketed product (Gangard®, 250** **mg) were given to the second group via gastric intubation. One milliliter of the normal saline was given to rabbits in the third group that was considered a negative control group. Rabbits were held in rabbit restrainers during blood sampling. Blood samples (0.5** **ml) were withdrawn in heparinized tubes from the ear vein at zero, 0.5, 1, 2, 3, 4, 6, 8, 12, and 24** **h after dosing. Blood samples were centrifuged at 4000** **rpm for 10** **min and were freezed at −20** **°C until further analysis. The GC concentrations in plasma samples were assayed according to Merodio et al.’s method (Vidyadhara et al., 2011).

### Chromatographic conditions and HPLC analysis of GC in plasma

GC was analyzed based on the method reported by Merodio et al. (2001). Briefly, Samples were chromatographed using a reversed-phase C18 column (250 × 4.6** **mm i.d.) which was guarded by a precolumn (4 × 4 mm i.d.). Acetonitrile (2%) in 0.05** **M acetate ammonium (adjusted at pH a 6.5) was used as a mobile phase, which was filtered through a 0.45-mm filter and degassed prior to the use (Vidyadhara et al., 2011). The flow rate was set at 1** **ml/min and the temperature at 38** **°C** **±** **2** **°C. The effluent was detected using a UV detector at 250** **nm. Acyclovir (AC) was used an internal standard (IS). The method was validated for accuracy, selectivity, and precision just prior to the beginning of the study.

### Pharmacokinetic data analysis

After gastric administration of the hard gelatin capsule loaded with the selected formula to New Zealand rabbits, a non-compartmental method was applied to construct the concentration versus time profile of GC. The maximum plasma concentration (C_max_) and the time of its occurrence (T_max_) were directly got from the concentration versus time curve. The area under the concentration versus time curve (AUC) and the mean residence time (MRT) were reported as measured. The relative bioavailability (Fr) with the reference product was calculated using the following equation:

Fr= (AUCtest/AUCref) * 100

### Statistical estimation of the results

All statistical analysis of the data was calculated by one way ANOVA via Minitab software (Minitab 19.1.1, Pennsylvania, United States), *p* value <.05 was considered significant. The adjusted determination coefficients (adjusted D2** **=** **0.5–1.0) were the criteria for the justification of the model chosen. The relationship between BPs and BQs was presented as contour plots and response surface plots made by changing XA and XB over the study range and setting the XC factor at its low and high levels. The *in vitro* drug release data *in vivo* pharmacokinetic parameters were represented as mean ± SE and analyzed using ANOVA analysis followed by with extended LSD post hoc test and *p* value less than .05 was considered significant.

## Results and discussions

### Characterization of the prepared NGCSD

The results of BQs for different prepared formulations have been expressed in Table 2. The effect of each process parameter (BPs) on each of the following: particle size, poly disperse index, zeta potential, entrapment efficiency percentage, drug content percentage, and the yield was calculated through the multinomial equations. The positive sign before the factor shows that the value increases and vice versa (Figure 2).

**Table 2.** Formulations code and BPS values of NGCSD particles as presented by 2^3^ full factorial design.

Formulae		F1	F2	F3	F4	F5	F6	F7	F8
Formula content code	CDX	0	1	0	1	0	1	0	1
	SHC	0	0	1	1	0	0	1	1
	GC	1	1	1	1	0	0	0	0
NGCSD particles characterization	PS (nm)	812.58 ± 35.09	699.10 ± 15.32	709.54 ± 28.25	654.89 ± 10.86	518.50 ± 13.80	288.50 ± 20.70	714.20 ± 23.14	423 ± 30.12
	PDI	0.72 ± 0.03	0.32 ± 0.05	0.66 ± 0.02	0.42 ± 0.4	0.51 ± 0.2	0.12 ± 0.01	0.53 ± 0.02	0.42 ± 0.03
	ZP (-mv)	11.25 ± 2.4	20.11 ± 2.5	10.60 ± 1.15	13.21 ± 2.15	22.33 ± 2.35	23.87 ± 2.27	12.74 ± 2.60	14.24 ± 3.25
	%Y	83.21 ± 2.1	82.10 ± 1.8	80.21 ± 2.5	88.10 ± 3.21	89.25 ± 1.2	94.23 ± 1.36	90.32 ± 2.31	88.12 ± 2.3
	%DC	84.33 ± 2.3	95.32 ± 3.1	92.47 ± 3.3	96.63 ± 3.5	85.13 ± 1.5	95.77 ± 2.1	93.11 ± 2.3	92.23 ± 3.7
	%EE	93.1 ± 2.1	94.3 ± 1.6	90.2 ± 3.1	95.2 ± 3.3	92.1 ± 2	96.2 ± 2.5	92.3 ± 2.3	91.2 ± 2.5

All the results are the average of three determinations ± SD.

### Particle size (PZ) and polydispersity index (PDI)

PZ and PDI affect both the drug release as well as bioavailability. The multinomial equations expressing these BQs are:

PZ=655.14–123.48A−42B+177.33C+88.44AB−20.50AC−128.56BC+63.37ABC

F=270.33, p<.001, and adjusted R2=0.9880

PDI=0.60–0.20A+0.06B+0.03C+0.07AB−0.04AC−0.03BC−0.02ABC

F=119.07, p<.001, and adjusted R2=0.9670

PZ equation shows that both CDX and SHC concentrations have a significant effect on the results. The highest value for CDX was +185.25, while +136.85 was the highest value for SHC however, GC concentration has the lowest positive coefficient on PZ (+32.22). Table 2 shows that the lowest and highest PZ was 288.50** **±** **20.70** **nm and 812.58** **±** **35.09** **nm for F6 and F1, respectively. Stimulatingly, CDX concentration in F6 was at its highest concentration while SHC concentration was low. The concentration of both CDX and SHC was minimal in F1. The results displayed that there was a converse relationship between particle size and polymer concentration with non-significant effect of GC concentration on the PZ. The results of PZ may be explained based on a smaller particle will be formed at low polymer concentration while an increase in particles growth was observed at higher concentration. Dudhipala et al. (2018) also reported in their investigation that the different types of drugs with different crystals need different concentrations of polymers to achieve uniform stable particle size (Merodio et al., 2001). Instinctively, both CDX and SHC concentrations (Xa and Xb) have a positive effect on the PDI value; while GC concentration (Xc) has no effect. The PDI values of all nano solid dispersions of GC were in the range of 0.12** **±** **0.01 and 0.72** **±** **0.03; which shows lower PDI values and a more uniform distribution in the particle size.

### Zeta potential (ZP)

One of the important parameters that is usually used for the characterization of the stability of nanoparticles is zeta potential. ZP measures the charge on the particles surface. It gives a good idea about the repulsion between particles with similar charges expresses the possibility of particles aggregation. It may be positive or negative, based on the chemistry of the particles. In general, the stability of the system increases with the increase in electrostatic repulsion between the particles. And the vice versa will be true, that’s the decrease in the ZP will lead to aggregation between the particles. Table 2 shows that all the formulations have a negative ZP ranging between 10.60** **±** **1.15 mv and 23.87** **±** **2.27 mv. This reveals an accepted repulsion between particles and a decrease in the possibility of aggregation.

Results show that F6, with a high concentration of CDX, experienced the highest ZP value (23.87** **±** **2.27mv). This can be attributed based on the − ve charge of the CDX hydroxyl groups. On the opposite side, the low − ve charge of SHC caused a significant decrease in ZP (F7).

### Yield percentage of GC solid dispersion nanoparticles (%Y)

Eight formulations of GC nanosolid dispersion were prepared by a solvent evaporation method with an accepted yield percentage ranged between 80.21** **±** **2.5 and 94.23** **±** **1.36 as shown in Table 2. The results were in agreement with Qushawy et al., and Sahoo et al., who have reported similar results for Glimpride solid dispersion production yield prepared by solvent evaporation technique (Sahoo et al., 2017; Alshehri et al., 2020).

### Drug content (DC%) of GC solid dispersion nanoparticles

The percentage of content for all formulations of GC nanosolid dispersion was calculated and represented in Table 2. The results show that the DC% ranged between 84.33** **±** **2.3 and 96.63** **±** **3.5%. The values of drug content support an appropriate selection of the carriers and preparation method. Sahoo et al. were reported results in a good accordance with our work findings (Sahoo et al., 2017).

The polynomial equation represent DC% parameter was as following

DC=31.11+14.18A+4.02B−6.51C+3.44AB+1.57AC−0.10BC+1.25ABC

F=66.64, p<.001, R2=0.9431

The equation reveals that only the concentration of both CDX and SHC affect drug content, while the GC showed a negative influence. Figure 1 shows the contour plot of the results. Previous study by Maghrabia et al. showed relevant results for ceftriaxone (Maghrabia et al., 2021).

### Entrapment efficiency (%EE) of GC solid dispersion nanoparticles

The entrapment efficiency of GC in solid dispersion was presented in Table 2, and expressed by the following equation:

%EE=36.52+20.66A+3.17B−8.82C+2.08AB−1.77AC−0.52BC+1.56ABC

F=84.4, p<.001, adjusted R2=0.975

**Figure 3.**
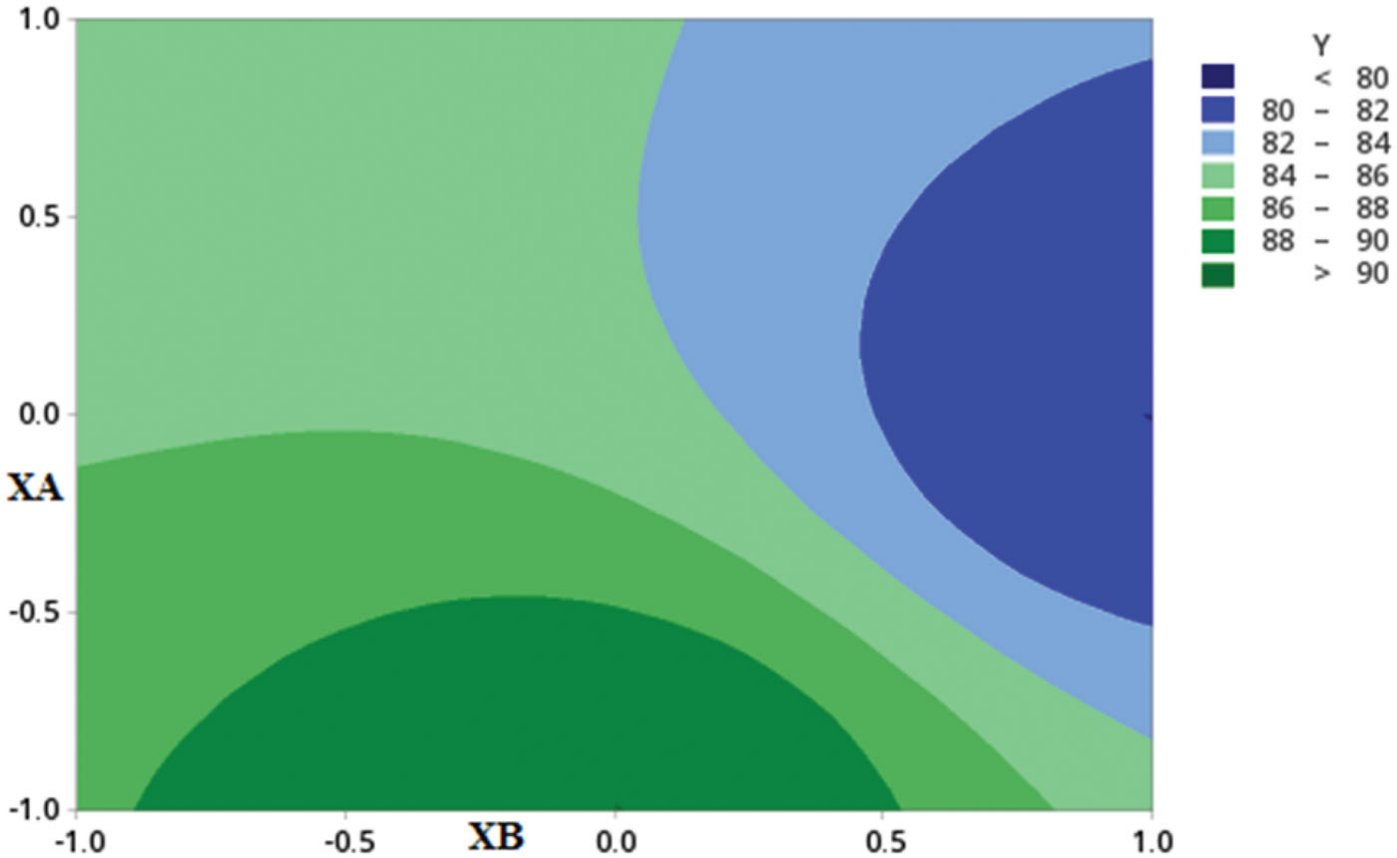
Contour plot showing the effect of CDX concentration (XA) and SHC concentration (XB)) on % EE.

This result also proves the ability of both CDX and SHC to trap GC into their channels and void spaces (Sahoo et al., 2017). Moreover, the contour plot (Figure 3) shows that high levels of CDX, with high or low levels of SHC and low levels of GC are preferred to produce NPs with high %EE (F6). Kawish et al. have conclude a related results for ceftriaxone nanoparticles (Alshehri et al., 2020).

### Micrometric flow properties studies of NGCSD formulations

The micrometric flow properties of the GC nano solid dispersion were studied by defining both the bulk and tapped density. The results were showed in Table 3 represent good micrometrics for the particles. That all formulae showed bulk and tapped density ranged between 0.35** **±** **0.06 − 0.49** **±** **0.04** **gm/cm^3^, and 0.40** **±** **0.04 − 0.58** **±** **0.03** **gm/cm^3^, respectively. These results were in good agreement with Chavan et al. and Qushawy et al. who prepared solid dispersions of nisoldipine and Glimepiride using a solvent evaporation technique (Alshehri et al., 2020; Chavan et al., 2020).

**Table 3.** The micrometric characterization of the nano GC solid dispersion formulations: bulk density, tapped density, Hausner’s ratio, Carr’s index, and angle of repose.

Formulation code	Bulk Density (g/cm^3^)	Tapped Density (g/cm^3^)	Hausenr’s Ratio	Carr’s Index (%)	Angle of Repose
F1	0.50** **±** **0.04	0.58** **±** **0.02	1.02** **±** **0.04	15.37** **±** **0.24	18.05** **±** **0.77
F2	0.40** **±** **0.03	0.53** **±** **0.04	1.13** **±** **0.02	11.20** **±** **0.44	19.16** **±** **0.72
F3	0.45** **±** **0.05	0.49** **±** **0.02	1.14** **±** **0.03	13.56** **±** **0.55	17.33** **±** **0.50
F4	0.41** **±** **0.03	0.55** **±** **0.03	1.02** **±** **0.09	14.33** **±** **0.08	18.99** **±** **0.44
F5	0.44** **±** **0.02	0.51** **±** **0.01	1.08** **±** **0.01	12.02** **±** **0.47	18.25** **±** **0.34
F6	0.42** **±** **0.02	0.56** **±** **0.03	1.09** **±** **0.03	17.08** **±** **0.22	19.14** **±** **0.55
F7	0.49** **±** **0.04	0.58** **±** **0.03	1.12** **±** **0.04	18.10** **±** **0.36	18.46** **±** **0.19
F8	0.35** **±** **0.06	0.40** **±** **0.04	1.16** **±** **0.03	13.95** **±** **0.12	18.67** **±** **0.42

Values were expressed in mean ± SD (*n*** **=** **3).

### Hausner’s ratio

The value of Hausner’s ratio is one of the parameters that indicate the flow properties of particles. According to USP the values less than 1.25 indicate a good (Chavan et al., 2020). As represented in Table 3, shows the Hausner’s ratio values of all prepared GC nanosolid dispersion formulations. It was in the range of 1.02** **±** **0.04 to 1.14** **±** **0.03, which indicated good accepted flowability for all formulations (Davis et al., 2018).

### The compressibility % (Carr’s index)

Micrometric references mention that there are an inverse relationship between the compressibility and the flowability properties of the particles (Chavan et al., 2020). The increase in Carr’s index, is expressed by a decrease in flowability. Carr’s index values between 5 and 12 indicate excellent flowability; the values between 12 and 16 show good flowability; the values ranged between 18 and 21 display fair flowability, and the values ranged from 23 to 35 display poor flowability (Browne et al., 2019). Results for all prepared GC nano solid dispersion formulations, showed that the value of Carr’s index was in the range of 11.20** **±** **0.44–18.10** **±** **0.36%, which indicated that the solid dispersion formulations ranged between a good and excellent flowability as represented in Table 3. The results were in an agreement with paracetamol solid dispersion flow properties results mentioned by Malviya et al. (2010).

### Angle of repose

The flowability of GC solid dispersion particles was also evaluated by the angle of repose test (Malviya et al., 2010). Excellent flowability is achieved when the angle of repose <20°. Values between 20–30° indicate good flowability, while the values between 30–34^°^ demonstrate passable flowability, and the angle of repose more than 34^°^ shows very poor flowability (Browne et al., 2019). The results shown in Table 3, reveals that all prepared GC nano solid dispersion displayed angle of repose in the range of 17.33** **±** **0.50 and 19.16** **±** **0.72 which indicated an excellent flowability of formulations and give a positive impression about its suitability for further capsules filling and other industrial processes (Browne et al., 2019).

## Characterization of the optimized formula NGCSD-F6

### DSC analysis of selected NGCSD formula

**Figure 4.**
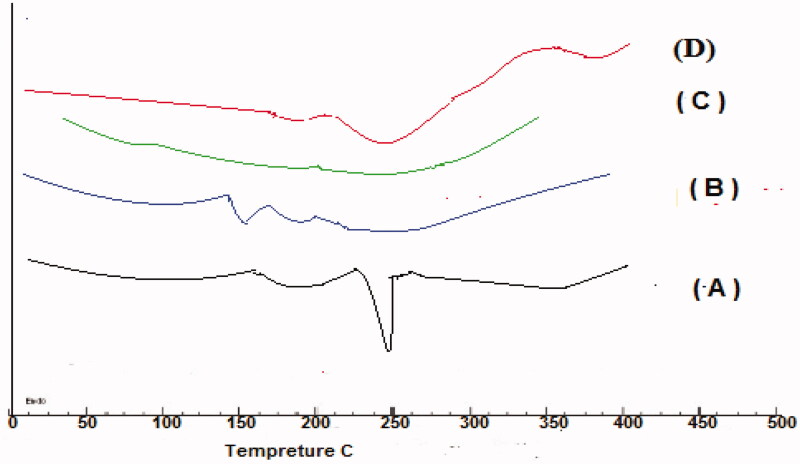
Differential scanning calorimetry (DSC) thermograms of pure GC (A), CDX (B), SHC(C), and optimized formula (D).

DSC method was used to study the interaction between GC, and the polymers used, DSC thermograms for each pure component, and the selected formulae are presented in Figure 4. A sharp distinctive endotherm was observed for GC at 252.13** **°C (Figure 4(A))

No endothermic peaks were observed for CDX and SHC due to their amorphous nature (Figure 4(B,C)). Figure 4(D) of the selected formula showed no shift in the characteristic peak of GC only broadening and decrease in the intensity was observed due to the decrease in concentration and polymer binding (Patel et al., 2016).

### FTIR spectroscopy studies

**Figure 5.**
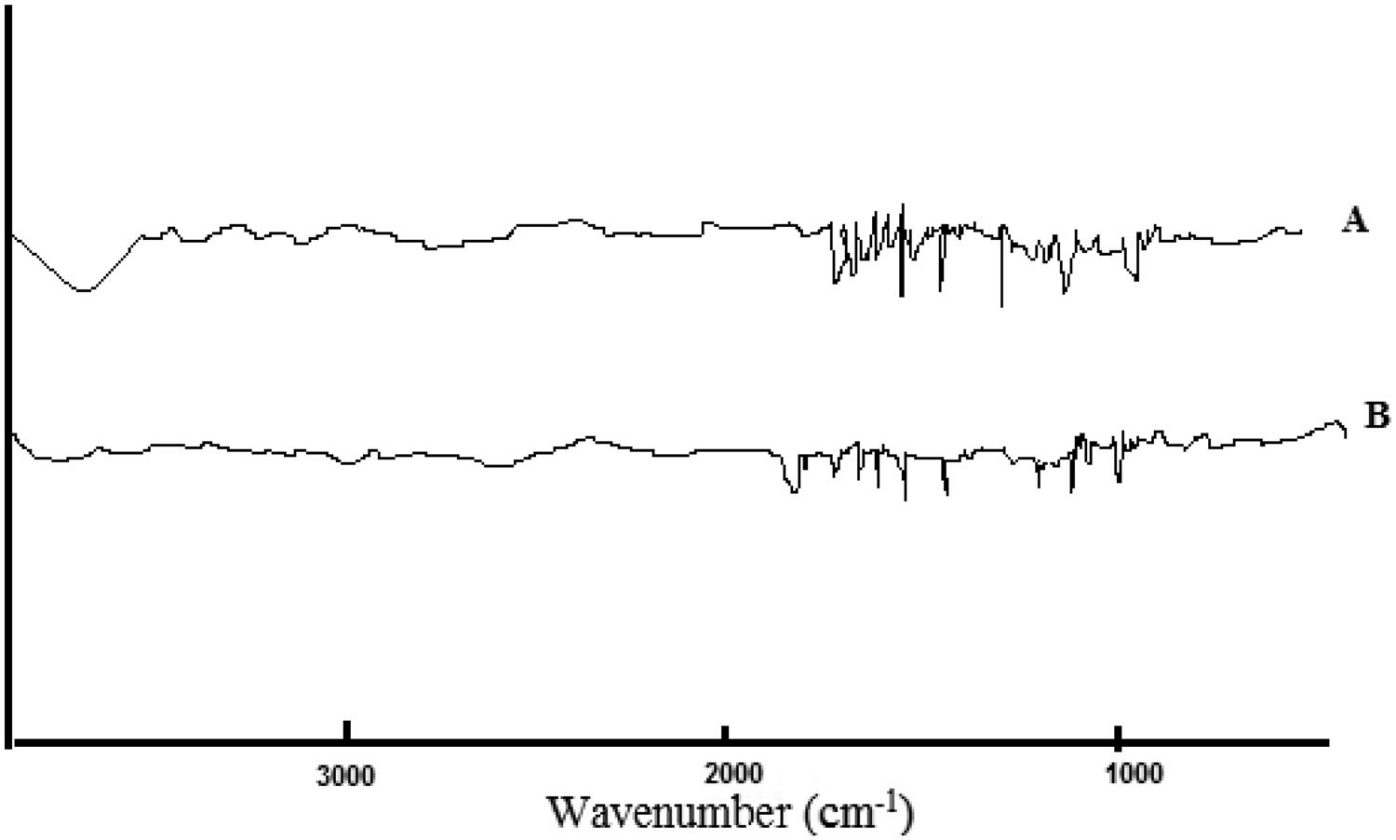
FTIR analysis of pure GC (A) and NGCSD-F6 (B).

FTIR method was conducted to identify drug/excipient interactions. Figure 5 shows the FTIR spectra of pure GC and the optimal solid dispersion formula of GC (F6).GC spectrum showed a peak at 3420** **cm^−1^ and 3433** **cm^−1^ which is characteristic to N-H/O-H, stretching, another absorption peak was observed at 1611** **cm^−1^, 1574** **cm^−1^, and 1543** **cm^−1^ which is characteristic to Aromatic C = C, others peaks at 1543** **cm^−1^, 1493** **cm^−1^, and 1097** **cm^−1^ which representing C = N, stretching, Aliphatic C–H, bending, and C–O–C, symmetric stretching, respectively. The spectra of the optimal formula showed broadening in the bands with no negative interactions between GC and the used polymers in the solid dispersions. Our results showed an accepted compatibility between the drug and used polymers (Märtson et al., 2021).

### Morphology study of optimal formula (NGCSD-F6) using scanning electron microscope (SEM)

**Figure 6.**
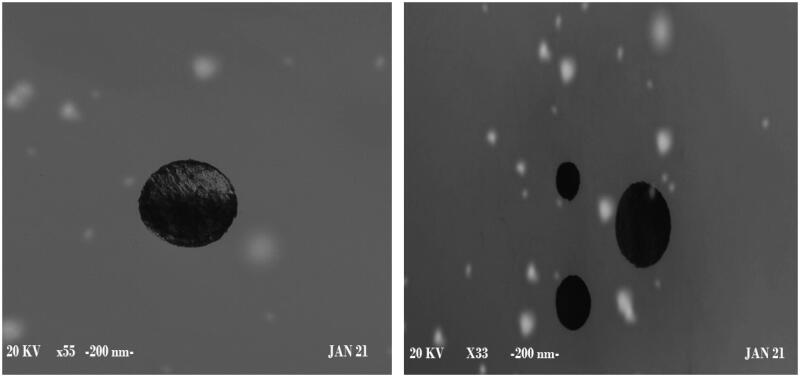
SEM image of selected formulae of NGCSD nanoparticles (F6).

Morphological inspection of the optimum formula (F6) was implemented. Figure 6 shows that the formula has a smooth sphere and encapsulates the drug with no irregularity or pits. This inveterate the formation of nanoparticles of CDX and SHC loaded with GC. The results prove the ionic gelation between the polymers and in situ encapsulation of GC (Yeung et al., 2020).

### Stability study

Table 4 shows that the selected formula of GC nano solid dispersion (F6) has good properties regarding particle size, zeta potential, and drug content percentage after storage for six months at 4°, 25°, and 40°. Also, it was noticed that ZP was decreased after one month of storage in the refrigerator which can be expressed based on decreasing the temperature may lead to a decrease in the charge on the particles. In addition, after one month of storage at 40** **°C, the particles showed darkening and shrinking in size which increased over time. This could be explained based on the loss of water and decomposition of drugs and polymers. In addition, all formulation were kept their good flowing properties during the six** **months and no significant increase Carr’s index was identified at the level of *p* ˂ .01.

**Table 4.** PS, PDI, ZP, DC%, and Carr’s index of NGCSD-F6, after storage at 4°, 25°, and 40° for six** **months.

	Conditions			
Time	Parameters	Refrigerator temperature	Room temperature 25** **±** **2 °C/40** **±** **5% RH	Accelerated stability 40** **±** **2 °C/75** **±** **5% RH
Zero	PS	288.5 ± 20.7	288.5 ± 20.7	288.5 ± 20.7
	PDI	0.12 ± 0.01	0.12 ± 0.01	0.12 ± 0.01
	ZP	23.87 ± 2.27	23.87 ± 2.27	23.87 ± 2.27
	DC%	95.77 ± 2.1	95.77 ± 2.1	95.77 ± 2.1
	Carr’s index	17.08 ± 0.22	17.08 ± 0.22	17.08 ± 0.22
1 month	PS	285.1 ± 10.7	283.5 ± 17.2	255.4 ± 25.2
	PDI	0.11 ± 0.03	0.13 ± 0.03	0.10 ± 0.02
	ZP	20.55 ± 3.15	24.15 ± 3.14	24.07 ± 3.33
	DC%	94.32 ± 3.0	95.08 ± 1.8	95.08 ± 3.1
	Carr’s index	18.28 ± 0.71	18.15 ± 0.24	17.11 ± 0.12
3 months	PS	284.0 ± 18.5	288.5 ± 20.7	248.3 ± 10.8
	PDI	0.14 ± 0.01	0.12 ± 0.01	0.09 ± 0.03
	ZP	19.77 ± 2.04	24.90 ± 2.59	25.66 ± 1.42
	DC%	94.28 ± 3.0	93.0 ± 2.1	93.07 ± 1.1
	Carr’s index	17.99 ± 0.14	17.85 ± 0.42	18.00 ± 0.13
6 months	PS	283.9 ± 17.8	288.5 ± 20.7	288.5 ± 20.7
	PDI	0.10 ± 0.05	0.12 ± 0.01	0.12 ± 0.01
	ZP	20.87 ± 1.44	25.37 ± 1.45	24.15 ± 3.02
	DC%	93.47 ± 3.0	94.57 ± 3.1	94.02 ± 1.1
	Carr’s index	18.28 ± 0.44	17.95 ± 0.31	18.10 ± 0.35

Results represented as a mean of three measurements ± SD.

### *In vitro* drug release from the optimized formula

**Figure 7.**
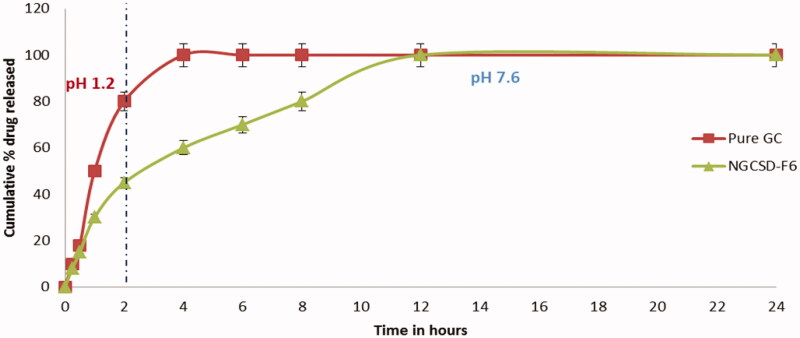
In vitro drug release of pure GC and NGCSD-FB in acidic media (pH 1.2) for 2** **h followed by alkaline media (pH 7.6) for next 22** **h.

The *in vitro* release study for both the pure GC and NGCSD-F6 were done in HCL buffer (pH1.2) for 2** **h followed by 22** **h in alkaline media (pH 7.6). The method was has minimum precision, recovery and dynamic range 62.11, 70.13, and 50.3, respectively. Pure GC showed burst release in acidic buffer and about 80.28** **±** **3.6% was detected after 2** **h and complete release of the drug was shown within 4 h (Figure 7). However, the selected formula expressed a completely different release pattern, the release pattern was bi-phase where an initial burst release was observed and about 53.23** **±** **5.5% was released within 50** **min followed by a regular release phase lasting for about 11** **h. The burst release could be explained based on the rapid dissolution of free drug adsorbed on the surface of solid dispersion particles. While the slower release phase (second phase) was due to the slow diffusion of GC from the prepared solid dispersion formula. Results reported by Kaushik et al. showed a sustained release of Glimepiride from solid dispersion particles (Kaushik & Pathak, 2018). In addition, similar results were reported for gliclazide solid dispersion particles by Febriyenti et al. (2019).

### Kinetic analysis of the GC release

The release data of the optimized formula was fitted to zero, first, Higuchi, and Korsmeyer–Peppas model. ‘R’ value was 0.885, 0.995, and 0.988 for first order, zero order and Higuchi’s kinetic model, respectively, with *n*** **=** **0.675. Based on results we concluded that both first, and Higuchi models are best fitted to describe the release kinetics of NGCSD-F6 with non- Fickian diffusion mechanism. Alves et al. reported the model of release was Higuchi model for efavirenz Solid dispersion (Alves et al., 2014).

### *In vivo* pharmacokinetic study

**Figure 8.**
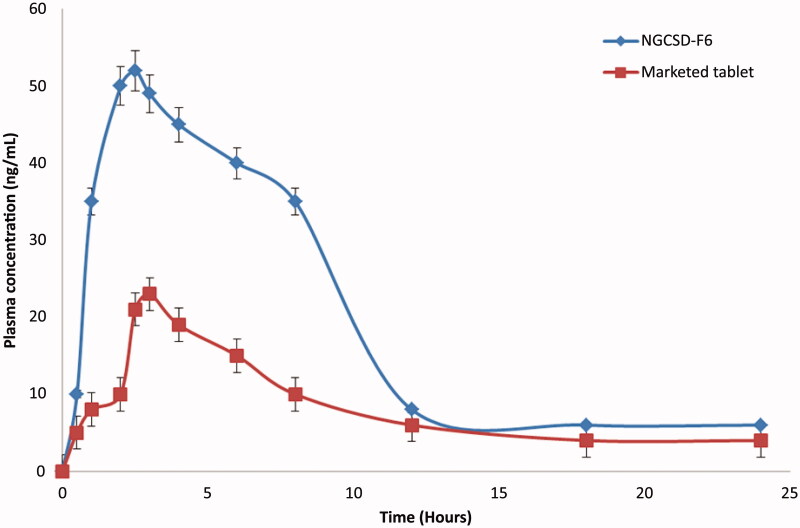
Plasma concentration time plot pf GC after oral administrations of NGCSD-F6 and marketed tablets in rabbits (mean ± SE).

Plasma levels data of GC after oral administration of NGCSD-F6 and commercial tablets showed that solid dispersion nanoparticles enhanced the bioavailability of GC compared with the control tablets. The absolute bioavailability for NGCSD-F6 and commercial tablets were 8.4% and 5.1%, respectively. Table 5 and Figure 8 reveal that the peak plasma concentrations of GC (C_max_) were 52.43** **±** **4.15 and 23.68** **±** **5.35** **ng/ml for NSDGC-F6 and marketed tablets, respectively. Results showed that a 2.56 fold increase in Cmax was observed for the selected formula compared with the commercial tablets. In addition, there was a significant increase (at p˂.01) in the area under the plasma concentration time plot of GC after oral administration of NGCSD-F6 (198.27** **±** **30.40** **ng/ml h) and the selected control tablets (89.94** **±** **33.86** **ng/ml h). The time to achieve the maximum concentration was 1.68** **±** **0.14 h for NGCSD-F6 while it showed 1.98** **±** **0.17 h for commercial tablets, this difference in tmax could be interpreted based on the increase in the permeability of GC in solid dispersion form compared with the control tablets. The study showed a 2.2 fold enhancement in the bioavailability of GC after oral administration of NGCSD particles. Results could be explained based on a decrease in the particle size and therefore the increase in the surface area. In addition, the formulation of GC as a solid dispersion lead to an increase in absorption and hence the bioavailability of GC (Das & Patra, 2017). The results in a full agreement with Sharma et al. who prepared carvedilol/chitosan nanoparticles for improving its bioavailability (Sharma et al., 2019).

**Table 5.** *In vivo* pharmacokinetics of GC following an oral administration of NGCSD-F6 and marketed tablets in rabbits.

In vivo pharmacokinetic parameter	NGCSD-F6	Commercial tablets
C_max_, ng/ml	52.43** **±** **4.15	23.68** **±** **5.35
T_max_, h	2.58** **±** **0.12	2.91** **±** **0.07
AUC_0-24_, ng/ml h	198.27** **±** **30.40	89.94** **±** **33.86
K_a_ h^−1^	3.9	2.1
MRT, h	7.6** **±** **0.1	6.9** **±** **0.2
Rel F	2.2	–

Results represented as a mean of three measurements ± SD.

## Conclusion

Nanoparticles solid dispersion encompassing GC with a different combination of CDX and SHC were successfully manipulated. The optimized formula showed good results regarding the yield, DC%, PZ, ZP, and flowing properties. *In vitro* release data of the optimized formula (NGCSD-F6) showed an accepted release over 12** **h. The bioavailability of GC after oral administration of optimized formula was 2.2 fold higher than the bioavailability of GC from convention tablets. The significant increase in bioavailability was interpreted based on two basic features; the first is the formulation of nanosize particles, and the second was the dispersion of the drug in a mixture of CDX and SHC polymers. Both parameters enhanced the drug absorption and increased the stability properties of the nanosolid dispersion formula. Finally, the NSD technique using natural polymers is an effective method for improving the absorption of class III BCS drugs.

## References

[CIT0001] Abarca RL, Rodríguez FJ, Guarda A, et al. (2016). Characterization of beta-cyclodextrin inclusion complexes containing an essential oil component. Food Chem 196:1836–75. 10.1016/j.foodchem.2015.10.023 26593579

[CIT0002] Alshehri S, Imam SS, Altamimi MA, et al. (2020). Enhanced dissolution of luteolin by solid dispersion prepared by different methods: physicochemical characterization and antioxidant activity. ACS Omega 5:6461–71. 32258881 10.1021/acsomega.9b04075 PMC7114142

[CIT0003] Alshehri S, Imam SS, Hussain A, et al. (2020). Potential of solid dispersions to enhance solubility, bioavailability, and therapeutic efficacy of poorly water-soluble drugs: newer formulation techniques, current marketed scenario and patents. Drug Deliv 27:1625–43. 33207947 10.1080/10717544.2020.1846638 PMC7737680

[CIT0004] Alves LDS, Soares MFDLR, De Albuquerque CT, et al. (2014). Solid dispersion of efavirenz in PVP K-30 by conventional solvent and kneading methods. Carbohydr Polym 104:166–74. 24607174 10.1016/j.carbpol.2014.01.027

[CIT0005] Asasutjarit R, Managit C, Phanaksri T, et al. (2020). Formulation development and in vitro evaluation of transferrin-conjugated liposomes as a carrier of ganciclovir targeting the retina. Int J Pharm 577:119084. 31988033 10.1016/j.ijpharm.2020.119084

[CIT0006] Bali GK, Singla S, Kashyap Y, et al. (2018). Preparation, physico-chemical characterization and pharmacodynamics of ceftriaxone loaded BSA nanoparticles. Nanomed Nanotech J 9:1–6.

[CIT0007] Browne E, Charifou R, Worku ZA, et al. (2019). Amorphous solid dispersions of ketoprofen and poly-vinyl polymers prepared via electrospraying and spray drying: a comparison of particle characteristics and performance. Int J Pharm 566:173–84. 31132449 10.1016/j.ijpharm.2019.05.062

[CIT0008] Chavan RB, Lodagekar A, Yadav B, Shastri NR. (2020). Amorphous solid dispersion of nisoldipine by solvent evaporation technique: preparation, characterization, in vitro, in vivo evaluation, and scale up feasibility study. Drug Deliv Transl Res 10:903–18. 32378174 10.1007/s13346-020-00775-8

[CIT0009] Davis MT, Potter CB, Walker GM. (2018). Downstream processing of a ternary amorphous solid dispersion: the impacts of spray drying and hot melt extrusion on powder flow, compression and dissolution. Int J Pharm 544:242–53. 29689366 10.1016/j.ijpharm.2018.04.038

[CIT0010] Das B, Patra S. (2017). Antimicrobials meeting the challenges of antibiotic resistance through nanotechnology. In: Nanostructures for antimicrobial therapy. Elsevier, 1–22.

[CIT0011] Desai MP, Labhasetwar V, Amidon GL, Levy RJ. (1996). Gastrointestinal uptake of biodegradable microparticles: effect of particle size. Pharm Res 13:1838–45. 8987081 10.1023/a:1016085108889

[CIT0012] Dudhipala N, Janga KY, Gorre T. (2018). Comparative study of nisoldipine-loaded nanostructured lipid carriers and solid lipid nanoparticles for oral delivery: preparation, characterization, permeation and pharmacokinetic evaluation. Artif Cells Nanomed Biotechnol 46:616–25. 10.1080/21691401.2018.1465068 29688077

[CIT0013] Febriyenti F, Rahmi S, Halim A. (2019). Study of gliclazide solid dispersion systems using PVP K-30 and PEG 6000 by solvent method. J Pharm Bioall Sci 11:262–7. 10.4103/jpbs.JPBS_87_18 PMC6662042 31555033

[CIT0014] Galar A, Valerio M, Catalán P, et al. (2021). Valganciclovir—ganciclovir use and systematic therapeutic drug monitoring. An invitation to antiviral stewardship. Antibiotics 10:77. 33467490 10.3390/antibiotics10010077 PMC7831032

[CIT0015] Ghosh S, Ghosh S, Sil PC. (2019). Role of nanostructures in improvising oral medicine. Toxicol Rep 6:358–68. 31080743 10.1016/j.toxrep.2019.04.004 PMC6502743

[CIT0016] Gill B, Kaur T, Kumar S, Gupta GD. (2010). Formulation and evaluation of glimepiride solid dispersion tablets. Asian J Pharm 4:112–8.

[CIT0017] Hong C, Wang J. (2018). Comparison of pharmaceutical quality of eight generic ganciclovir injections in China and Cymevene. J Chemother 30:310–5. 30843773 10.1080/1120009X.2018.1516271

[CIT0018] Jaglal Y, Osman N, Omolo CA, et al. (2021). Formulation of pH-responsive lipid-polymer hybrid nanoparticles for co-delivery and enhancement of the antibacterial activity of vancomycin and 18β-glycyrrhetinic acid. J Drug Deliv Sci Technol 64:102607.

[CIT0019] Kamalakkannan V, Puratchikody A, Ramanathan L. (2013). Development and characterization of controlled release polar lipid microparticles of candesartan cilexetil by solid dispersion. Res Pharm Sci 8:125–36. 24019822 PMC3764676

[CIT0020] Kaushik S, Pathak K. (2018). Solubility enhancement of glimperide: development of solid dispersion by solvent melt method, characterization and dosage form development. Pharm Biomed Res 3:1–13.

[CIT0021] Kiroula N, Negi J. (2016). Preparation and characterization of ganciclovir loaded glutathione modified gold nanoparticles. Pharm Sci 78:117.

[CIT0022] Limmatvapirat S, Panchapornpon D, Limmatvapirat C, et al. (2008). Formation of shellac succinate having improved enteric film properties through dry media reaction. Eur J Pharm Biopharm 70:335–44. 18430548 10.1016/j.ejpb.2008.03.002

[CIT0023] Maghrabia A, Boughdady M, Meshali M. (2021). Design and optimization of new enteric nanoparticles of ceftriaxone for oral delivery: in vitro and in vivo assessments. Int J Nanomed 16:5937–53. Aug 28 10.2147/IJN.S319176 PMC8414076 34511899

[CIT0024] Maghrabia AE, Boughdady MF, Meshali MM. (2019). New perspective enteric-coated tablet dosage form for oral administration of ceftriaxone: in vitro and in vivo assessments. AAPS PharmSciTech 20:306–12. 31512022 10.1208/s12249-019-1512-y

[CIT0025] Maheen S, Rasul A, Hanif M, Khan HU. (2020). Lipospheres for simultaneous controlled release and improved pharmacokinetic profiles of saxagliptin-enalapril: formulation, optimization, and comparative in vitro-in vivo evaluation. AAPS PharmSciTech 21:1–16. 10.1208/s12249-020-01733-w 32651896

[CIT0026] Malviya R, Srivastava P, Bansal M, Sharma PK. (2010). Improvement of dissolution behavior of paracetamol using solid dispersion technique. Int J Pharm Sci Res 1:95–9.

[CIT0027] Märtson A, Edwina A, Burgerhof J, et al. (2021). Ganciclovir therapeutic drug monitoring in transplant recipients. J Antimicrob Chemother 76:2356–63. 34160036 10.1093/jac/dkab195 PMC8361328

[CIT0028] Mehta A, Vasanti S, Tyagi R, Shukla A. (2009). Formulation and evaluation of solid dispersions of an anti-diabetic drug. Curr Trends Biotechnol Pharm 3:76–84.

[CIT0029] Merodio M, Arnedo A, Renedo MJ, Irache JM. (2001). Ganciclovir-loaded albumin nanoparticles: characterization and in vitro release properties. Eur J Pharm Sci 12:251–9. 11113644 10.1016/s0928-0987(00)00169-x

[CIT0030] Noolkar SB, Jadhav N, Bhende SA, Killedar SG. (2013). Solid-state characterization and dissolution properties of meloxicam-moringa coagulant-PVP ternary solid dispersions. AAPS PharmSciTech 14:569–77. 23483432 10.1208/s12249-013-9941-5 PMC3665984

[CIT0031] Öztürk-Atar K, Eroğlu H, Gürsoy R, Çaliş S. (2019). Current advances in nanopharmaceuticals. J Nanosci Nanotechnol 19:3686–705. 30764926 10.1166/jnn.2019.16764

[CIT0032] Patel R, Gajra B, Parikh RH, Patel G. (2016). Ganciclovir loaded chitosan nanoparticles: preparation and characterization. J Nanomed Nanotechnol 7:411.

[CIT0033] Pathak K, Pattnaik S. (2018). Stability testing parameters and issues for nano technology-based drug products. In: Methods for stability testing of pharmaceuticals. Springer, 293–305.

[CIT0034] Raza A, Ngieng SC, Sime FB, et al. (2021). Oral meropenem for superbugs: challenges and opportunities. Drug Discov Today 26:551–60. 33197621 10.1016/j.drudis.2020.11.004

[CIT0035] Reginald-Opara JN, Attama A, Ofokansi K, et al. (2015). Molecular interaction between glimepiride and Soluplus®-PEG 4000 hybrid based solid dispersions: characterisation and anti-diabetic studies. Int J Pharm 496:741–50. 26581773 10.1016/j.ijpharm.2015.11.007

[CIT0036] Ricarte RG, Van Zee NJ, Li Z, et al. (2019). Recent advances in understanding the micro- and nanoscale phenomena of amorphous solid dispersions. Mol Pharm 16:4089–103. 31487183 10.1021/acs.molpharmaceut.9b00601

[CIT0037] Sahoo AC, Kanungo SK, Dinda SC, et al. (2017). Improvement in micromeritic properties and dissolution rate of glimepiride. WJPR 6:1545–60.

[CIT0038] Sarbajna RM, Preetam A, Devi AS, et al. (2011). Studies on crystal modifications of ganciclovir. Mol Cryst Liq Cryst 537:141–54.

[CIT0039] Shaikh K, Patwekar S, Payghan S, D’Souza J. (2011). Dissolution and stability enhancement of poorly water soluble drug-lovastatin by preparing solid dispersions. Asian J Biomed Pharm Sci 1:24–31.

[CIT0040] Sharma M, Sharma R, Jain DK, Saraf A. (2019). Enhancement of oral bioavailability of poorly water soluble carvedilol by chitosan nanoparticles: optimization and pharmacokinetic study. Int J Biol Macromol 135:246–60. 31128197 10.1016/j.ijbiomac.2019.05.162

[CIT0041] Soni L, Ansari M, Thakre N, et al. (2017). Development and in-vitro evaluation of furosemide solid dispersion using different water soluble carriers. Int J Res Dev Pharm Life Sci 6:2571–5.

[CIT0042] Ting JM, Porter IWW, Mecca JM, et al. (2018). Advances in polymer design for enhancing oral drug solubility and delivery. Bioconjug Chem 29:939–52. 29319295 10.1021/acs.bioconjchem.7b00646

[CIT0043] Tran P, Pyo YC, Kim DH, et al. (2019). Overview of the manufacturing methods of solid dispersion technology for improving the solubility of poorly water-soluble drugs and application to anticancer drugs. Pharmaceutics 11:132. 30893899 10.3390/pharmaceutics11030132 PMC6470797

[CIT0044] Vidyadhara S, Babu JR, Sasidhar RLC, et al. (2011). Formulation and evaluation of glimepiride solid dispersions and their tablet formulations for enhanced bioavailability. Pharmanest 1:15–20.

[CIT0045] Wallace SJ, Li J, Nation R, et al. (2012). Drug release from nanomedicines: selection of appropriate encapsulation and release methodology. Drug Deliv Transl Res 2:284–92. 23110256 10.1007/s13346-012-0064-4 PMC3482165

[CIT0046] Wang Q, Sun C, Xu B, et al. (2018). Synthesis, physicochemical properties and ocular pharmacokinetics of thermosensitive in situ hydrogels for ganciclovir in cytomegalovirus retinitis treatment. Drug Deliv 25:59–69. 29228826 10.1080/10717544.2017.1413448 PMC6058567

[CIT0047] Yeung A, Souto E, Durazzo A, et al. (2020). Big impact of nanoparticles: analysis of the most cited nanopharmaceuticals and nanonutraceuticals research. Curr Res Biotechnol 2:53–63.

